# Development of the Triboelectric Nanogenerator Using a Metal-to-Metal Imprinting Process for Improved Electrical Output

**DOI:** 10.3390/mi9110551

**Published:** 2018-10-27

**Authors:** Moonwoo La, Jun Hyuk Choi, Jeong-Young Choi, Taek Yong Hwang, Jeongjin Kang, Dongwhi Choi

**Affiliations:** 1 Molds & Dies Technology R&D Group, Korea Institute of Industrial Technology (KITECH), Incheon 21999, Korea; mla@kitech.re.kr (M.L.); wjddud21@kitech.re.kr (J.-Y.C.); taekyong@kitech.re.kr (T.Y.H.); doublej@kitech.re.kr (J.K.); 2 Department of Mechanical Engineering, Kyung Hee University, Yongin 17104, Korea; cjng123@khu.ac.kr

**Keywords:** triboelectric nanogenerator, nanoimprinting, nanostructures, microstructures, femtosecond laser

## Abstract

Triboelectric nanogenerators (TENG), which utilize contact electrification of two different material surfaces accompanied by electrical induction has been proposed and is considered as a promising energy harvester. Researchers have attempted to form desired structures on TENG surfaces and successfully demonstrated the advantageous effect of surface topography on its electrical output performance. In this study, we first propose the structured Al (SA)-assisted TENG (SA-TENG), where one of the contact layers of the TENG is composed of a structured metal surface formed by a metal-to-metal (M2M) imprinting process. The fabricated SA-TENG generates more than 200 V of open-circuit voltage and 60 µA of short-circuit current through a simple finger tapping motion. Given that the utilization of the M2M imprinting process allows for the rapid, versatile and easily accessible structuring of various metal surfaces, which can be directly used as a contact layer of the TENG to substantially enhance its electrical output performance, the present study may considerably broaden the applicability of the TENG in terms of its fabrication standpoint.

## 1. Introduction

The development of portable electronic devices has received considerable attention due to their unique advantages such as convenience and excellent functionality, which has enhanced the lives of people in various aspects. These devices require power without using wires from an external power source. As a result, energy-harvesting technologies, which convert the available sustainable energies into electricity has also attracted attention worldwide [1]. The eminent concept of the triboelectric nanogenerator (TENG), which utilizes contact electrification of two different material surfaces accompanied by electrical induction, has been proposed [2]. Since its first proposal in 2012, TENG has been actively studied, hence, it is considered to be a promising energy harvesting technology to operate portable electronic devices without spatio-temporal limitations on the basis of its advantages, such as material selection diversity, high efficiency and high shape adaptability [3,4,5,6,7,8]. The most actively conducted research topic in the field of the TENG is the enhancement of its electrical output performance, which is similar to those of other energy-harvesting technologies [9]. Given that the fundamental operating mechanism of the TENG is based on the contact and separation between two surfaces, the simplest and most widely used strategy to enhance the electrical output performance is the introduction of micro- and nanoscale structures onto the surfaces where friction occurs [3,5,10,11,12]. The formation of the surface structures significantly increases local contact pressure resulting in further increases in the contact area between two contact surfaces, thereby generating a high amount of electrical charge on the contact surface [13]. Given that the most widely utilized material is based on polymer, many researchers have attempted to form desired structures onto polymer surfaces through various subtractive and additive fabrication methodologies. These researchers have successfully demonstrated the benefits of surface topography on the electrical output performance of the TENG. Although the electrical output performance of the TENG has drastically increased due to these efforts, the introduction of structures on the polymer surface accelerates the mechanical wear of the surface of the friction due to the increased local contact pressure. Considering that the operation of a TENG is based on the friction between two surfaces, one of the major issues to be resolved is the problem of mechanical wear [5,14,15]. However, the introduction of structures onto the polymer has relatively weak mechanical characteristics compared with other engineering materials, which is definitely unfavorable in terms of mechanical wear. A potential strategy to reduce mechanical wear in the TENG can be the formation of the structures on metal surfaces instead of polymer surfaces. Metal is a widely utilized engineering material with better mechanical characteristics, thereby resulting in higher abrasion resistance than those of the polymer. Metal is also widely utilized in the TENG causing friction with the polymer surface. The common approach to the fabrication of small-scale structures on metal is first to fabricate a prepatterned polymer and use it as a sacrificial layer. Then, subsequent metal deposition on its surface followed by lift-off or an etching process, which are considered to be complex and labor intensive multistep processes due to their requirement for difficult processing conditions [16,17]. Consequently, most TENGs, which utilize a polymer-metal friction to generate electrical output performance, only have a structure on the polymer surface because it takes considerable effort to form structures on the metal surface than on the polymer surface [3]. Hence, the proposal of facile and highly accessible strategies to form structures onto the metal surface will open another horizon to enhance the electrical output performance of the TENG.

In this study, we propose a structured Al (SA)-assisted TENG (SA-TENG), where one of the contact surfaces of the TENG is composed of the structured metal surface formed by a metal-to-metal (M2M) imprinting process. The imprinting process is a low-cost and rapid process that involves transcribing structures onto the substrate of interest by using a stamp. The present study utilizes a precise femtosecond (fs) laser to fabricate steel stamps, which have optically induced micro- and nanoscale patterns and these patterns are transcribed on Al substrates by applying heat and pressure. The M2M imprinting process is optimized by applying a systematic approach. As a result, conical microstructures and line nanostructures are successfully transcribed onto the Al substrates. The structures on the Al substrates considerably enhance the electrical output performance of the present SA-TENG. Given that the utilization of the M2M imprinting process allows the rapid, versatile and easily accessible structuring of the metal substrate, which can be directly used as a contact layer on the TENG to significantly enhance its electrical output performance, the present study may significantly broaden the applicability of the TENG in terms of its fabrication standpoint.

## 2. Materials and Methods

### 2.1. Fabrication of Stamps via fs Laser Processing of Steels

The laser used for stamp fabrication was a Ti:sapphire fs laser system on the basis of chirped pulse amplification. This laser system generated 120 fs pulses with a maximum energy of 5 mJ/pulse and operated at a central wavelength of 800 nm with a repetition rate of 1 kHz. Prior to fs laser processing, we polished steel plates mechanically by using 80 nm-grade colloidal silica until the average surface roughness (Ra) reached 4.1 nm [18]. This polishing process can minimize non-uniform laser energy absorption at the surface due to scratches and pre-existing surface structures [19].

By irradiating the steel plates with fs laser pulses, two types of stamps each with laser-induced conical microstructures and periodic line nanostructures were fabricated on steel (STAVAX, ASSAB, Incheon, South Korea). To produce conical microstructures, circularly polarized fs laser pulses were used so that each conical microstructure tended to form circularly at the steel surface [20] and the pulses were weakly focused onto the surface with a 1/e^2^ intensity spot radius of 270 μm at a fluence of 0.37 J/cm^2^. The speed of raster scanning was 2 mm/s and the distance between scanning lines was 30 μm. On the other hand, to create periodic line nanostructures, linearly polarized fs laser pulses were used, since the orientation (wave vector) of periodic line nanostructures is parallel to laser polarization [21]. Compared to the case of conical microstructure formation, fs laser pulses were rather tightly focused with a 1/e^2^ intensity spot radius of 90 μm. Additionally, a lower laser fluence, faster raster scanning speed and larger scanning line distance of 0.23 J/cm^2^, 12 mm/s and 40 μm were used for the fabrication of periodic line nanostructures. All experiments in this sub-section were performed in ambient air at normal incidence.

### 2.2. The SA Fabrication by M2M Imprinting Process

SA fabrication is based on the M2M imprinting process. An as-fabricated steel stamp was utilized and the conventional Al plate was first electropolished and then utilized as a substrate in the M2M imprinting process. The Al substrate and the steel stamp with structures to be transcribed were vertically stacked, and appropriate pressure and heat were applied using a conventional hot-pressing machine (QM900, QMESYS, Gunpo, South Korea).

### 2.3. Measurement of the Electrical Output Performance of the TENG

A vertical cyclic force was applied on the contact surfaces of the TENG by using the conventionally available vibration testing machine (KD-9363ED-41E, Kingdesign, New Taipei City, Taiwan). The open circuit voltage (*V*_OC_), which assumed the infinite load resistance connected to the TENG, was measured by directly connecting the oscilloscope (DS1074z, Rigol, Beaverton, OR, USA) equipped with the high voltage probe (DP-50, Pintek, New Taipei City, Taiwan) to the TENG. The internal resistances of the oscilloscope and the high voltage probe were 10 MΩ and 54 MΩ, respectively. The short circuit current (*I*_SC_) was measured with a low-noise current preamplifier (SR570, Stanford Research Systems, Sunnyvale, CA, USA) that was connected with an oscilloscope.

## 3. Results

### 3.1. Fabrication of the SA

**Figure 1:**
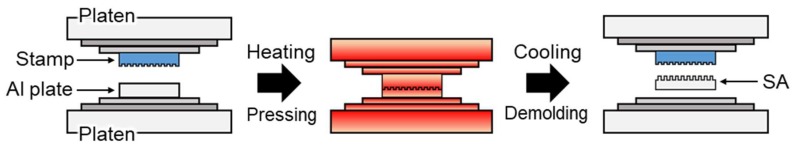
A schematic illustration of the metal-to-metal (M2M) imprinting process. An as-prepared steel stamp and the Al plate is vertically stacked between two platens. Heating and pressing induces transcription of the structures onto the Al plate and following this, cooling and demolding enables detachment of the structured Al (SA) from the stamp.

Figure 1 shows the SA fabrication procedure, which is composed of two steps, as follows: (1) the fabrication of the high-strength steel stamp with structures using a fs laser and (2) as-fabricated stamp utilization to transcribe structures on the Al substrate by using the M2M imprinting process. Since its development, the fs laser has become one of the most promising tools for direct surface patterning on various engineering materials, including metals. Many reports have shown that the interaction between the laser spot and metal surfaces induces natural consequences with the production of nano- to microstructures on the metal surface. These structures also modify the surface properties [17,18,19,20,21]. According to the literature, steel substrates are utilized as engineering materials, nano- and microstructures are easily formed and then as-prepared steel plates are directly utilized as metal stamps in the M2M imprinting process [17]. The M2M imprinting process directly transcribes the structures onto the steel stamp and then to the other metal substrates by applying heat and pressure. In this study, considering that the stamp was composed of steel, which has relatively high strength, most of the other conventionally available metals, such as Al, Cu, Ag and Au, can be utilized as a target substrate. Here, considering that Al is often utilized as one of the contact layers of the TENG due to its low electron affinity, we exclusively utilizes the Al plate as our substrate, where the transcription of the structures occurred. After placing the steel stamp and the Al plate up and down, heat and pressure were applied using a hot-pressing machine. Then, the patterns on the stamp was transcribed onto the Al plate.

**Figure 2:**
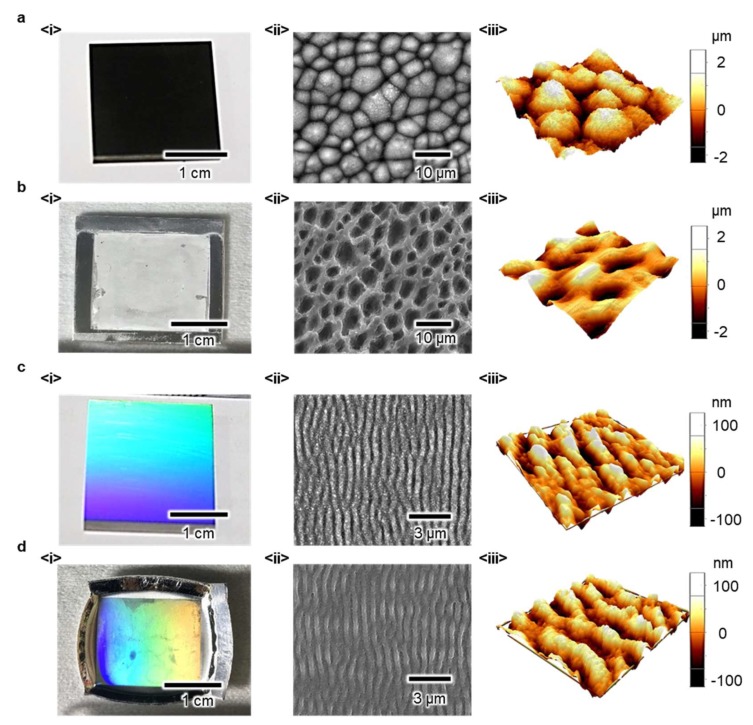
Images and investigation results of the as-prepared steel stamp and the imprinted SA. (**a**) (i) real, (ii) scanning electron microscopic (SEM) and (iii) atomic force microscopy (AFM) images of the steel stamp possessing conical microstructures, (**b**) (i) real, (ii) SEM and (iii) AFM images of the SA with microstructures, (**c**) (i) real, (ii) SEM and (iii) AFM images of the steel stamp possessing line nanostructures, (**d**) (i) real, (ii) SEM and (iii) AFM images of the SA with line nanostructures.

Figure 2a-(i) shows the images of the fabricated steel stamp with conical microstructures on its surface. The processing conditions to form these structures were based on the literature on the formation of laser-induced microstructures on metal surfaces. The details are provided in the Materials and Methods section. The scanning electron microscopic (SEM, FEI Quanta 200F, ThermoFisher Scientific, MA, USA) image as shown in Figure 2a-(ii) demonstrates the successful formation of conical microstructures on the steel stamp by using the fs laser. Prior to the M2M imprinting process, the features on the stamp surface were investigated using atomic force microscopy (AFM, Park XE-100, Park Systems, Suwon, South Korea) for quantitative analyses, as shown in Figure 2a-(iii). Three representative parameters, that is, width (*W*), period (*P*) and height (*H*) of the structures describing the features on the surface were measured and then utilized to investigate the effect of the processing conditions of the M2M imprinting on the transcription qualities (*TQ*s). This is shown as follows:

(1) $$TQ_{W}=\;\frac{W_{\mathrm{substrate}}}{W_{\mathrm{stamp}}},\;TQ_{P}=\;\frac{P_{\mathrm{substrate}}}{P_{\mathrm{stamp}}},\;TQ_{H}=\;\frac{H_{\mathrm{substrate}}}{H_{\mathrm{stamp}}}$$

In general, the imprinting pressure (*P*_i_), imprinting temperature (*T*_i_), and holding time (*t*_i_) are utilized as the control parameters of the M2M imprinting process. *TQ*s are investigated by varying these parameters, hence, one of the conditions (*P*_i_ = 15 MPa, *T*_i_ = 200 °C, *t*_i_ = 1 min), where the features of conical microstructures are transcribed well onto the Al substrate, is determined (see Table S1–S3 in Supplementary Materials). Figure 2b-(i–iii) show the structured Al substrate, SEM and AFM images, respectively.

To show the ability of the M2M imprinting process for nanoscale structure transcription, we fabricated the steel stamp possessing periodic line nanostructures. The fabrication procedures were based on a previous study and is also described in the Section 2. As shown in Figure 2c-(i), the size of the features on the stamp is comparable with the range of the period of the visible lights and the stamp exhibits a structural color. The SEM images shown in Figure 2c-(ii) show that the laser-induced periodic line nanostructures were formed well on the steel stamp surface. Experiments and analyses in terms of *TQ*s were conducted in a similar manner to the experiment above. Thus, the condition (i.e., *P*_i_ = 25 MPa, *T*_i_ = 200 °C, *t*_i_ = 5 min) where the features of line nanostructures were transcribed on the Al substrate well were determined (see Table S4 in Supplementary Materials). As shown in Figure 2d-(i), the transcribed nanoscale structures on the Al substrate showed structural color. This result directly showed that the present M2M process could be applied to form a variety of patterns in nanometer and micrometer scales on the Al substrate.

### 3.2. Utilization of the SA and Resultant Enhanced Electrical Output Performance of the TENG

**Figure 3:**
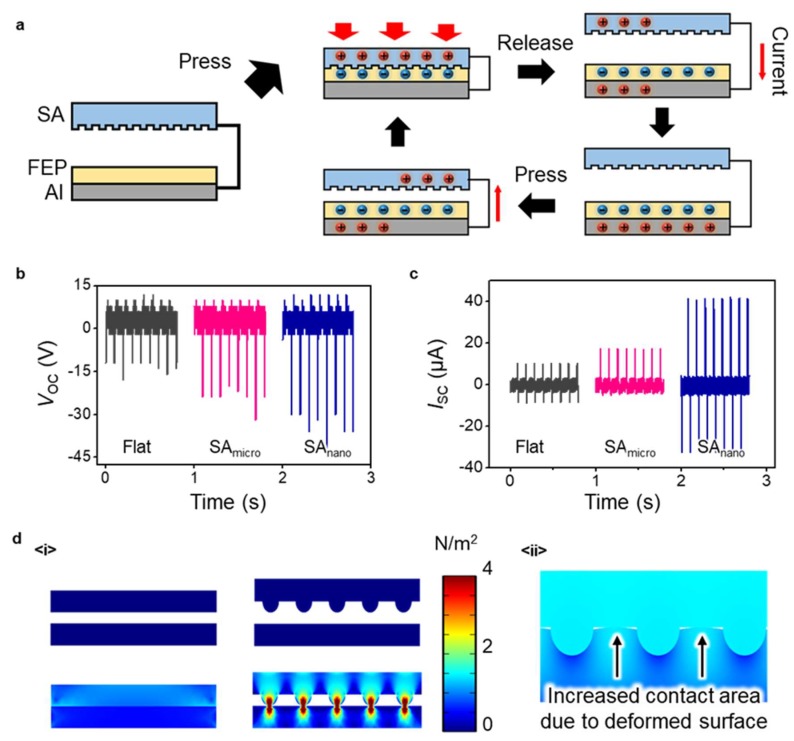
Schematics of the operating mechanism of the SA-TENG and its electrical output performance. (**a**) Operating mechanism of the SA-TENG. Successive contact and separation between FEP and Al surfaces generates electrical charges and corresponding electric current flows, (**b**) comparison of the V_OC_s and (**c**) I_SC_s generated from the TENGs with flat Al, SA_micro_ and SA_nano_,(**d**) (i) numerical analysis showing the increased local contact pressure on the structured surfaces, (ii) the deformation of the surface due to the increased local contact pressure which increases the total contact area, resulting in an increase in the amount of electrical charges generated on the surface.

As-prepared SA possessing micro- and nanoscale structures are utilized as one of the contact layers of the TENG. The electricity generation mechanism of the TENG is shown in Figure 3a. The counter contact layer of the SA is composed of conventional fluorinated ethylene propylene (FEP) film that is attached to the Al film. Given the difference in the electron affinity between FEP and Al, contact spontaneously generates positive and negative electrical charges on Al and FEP, respectively. The separation of the two surfaces induces generation of the net electrical charges on both surfaces and corresponding charge generation on the FEP film surface induces the additional positive charges on the Al film beneath the FEP film. During this process, the electrical current, which is generated from the movement of the electrical charges, flows. Another contact of Al with FEP satisfies the electro-neutrality between the electrical charges on Al and FEP. Hence, electrical current flows in the opposite direction. Consequently, repetitive contact and separation between Al and FEP result in alternating current, as shown in Figure 3a.

To investigate the effect of the features on the electrical output performance of the TENG, we utilized flat Al (control), SA with conical microstructures (SA_micro_) and SA with line nanostructures (SA_nano_) for comparison. Under the same experimental conditions, the *V*_OC_s and the *I*_SC_s of each case were measured. Vertical cyclic force with a magnitude of 8 N and a frequency of 10 Hz was applied on the contact surfaces by using the conventionally available vibration testing machine.

As shown in Figure 3b,c, both *V*_OC_s and *I*_SC_s generated from SA_micro_ and SA_nano_ are larger than those from the control. The close-up views of the *V*_OC_ and *I*_SC_ show the detailed profiles of the peaks (see Figure S1 in Supplementary Materials). The result in Figure 3b,c is consistent with the previously reported findings with the various TENGs possessing surface structures. The structures on the surface are known to increase the local pressure during contact situations resulting in further increases in the contact area between two contact surfaces, thereby increasing the amount of electrical charges generated on the surface [13]. The amount of electrical charges on the surface is positively correlated with the electrical output performance of the TENG. This claim can be verified using the numerical simulation shown in Figure 3d. To investigate the effect of the surface structures on the developed localized pressure during contact, we conducted a numerical simulation by using the commercial software COMSOL Multiphysics^TM^ (COMSOL Inc., Burlington, MA, USA). The simplified models of the flat and structured Al surfaces are shown in Figure 3d-(i). When we apply a force, the highly increased local contact pressure can be observed on the structured surface compared to that on the flat surface. This increased local contact pressure further induces deformation of the contact surface, resulting in an increase in the contact area between two surfaces as shown in Figure 3d-(ii). Details on the numerical analysis of the surface deformation are in the Figure S2 in Supplementary Materials. The result directly supports the claim that the SA is preferable in enhancing the electrical output performance of the TENG due to the presence of the structures on its surface.

Considering that metal is a widely utilized engineering material with better mechanical characteristics with higher endurance than those of the polymer, one of the benefits of using the structured metal surface as one of the layers of the TENG would be the stable electrical output performance. To support such a claim, the degradation behavior of the electrical output performance as well as the endurance of the structures on the present SA under an extremely large number of the contact separation working cycles were investigated. It is noteworthy to mention that there is no noticeable degradation of the electrical output performance and significant change of the surface structures after ~10^6^ working cycles (see Figure S3 in Supplementary Materials). The experimental results directly showed that the formation of the structures on metal in the present study could be a potent strategy not only to enhance the electrical output performance of the TENG, but also to increase the endurance of the device.

### 3.3. Fabrication of the SA-TENG

**Figure 4:**
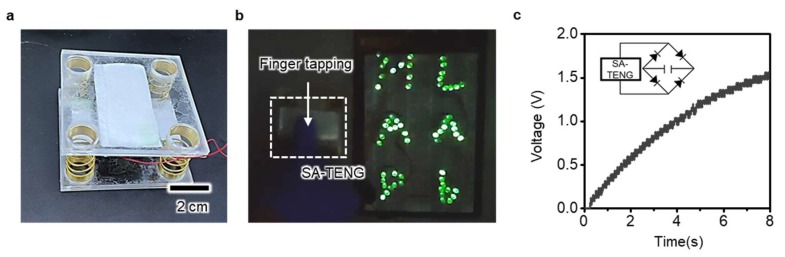
A fabricated SA-TENG and its demonstration of operation. The electrical output generated from the SA-TENG by a simple finger tapping motion enables simultaneous lighting up of the tens of LEDs. Applying the vertical cyclic force on the device charges the conventional capacitor.

On the basis of the results in the previous section, we fabricated the SA-TENG, which included a counter layer composed of FEP and Al films in the previous experiment via SA_nano_ and as shown in Figure 4a. The FEP film attached onto the Al film and as-prepared SA_nano_ were utilized as two separated components of the SA-TENG, which contacted and separated with each other to generate electricity. The two components were attached and supported by the polydimethylsiloxane-attached poly(methyl methacrylate) plate, which was elaborately carved by the laser cutter. Four compressive springs located at the vertices of the rectangular plate maintained the position of the components with a separated status through spring resilience. When we pressed the upper side of the SA-TENG, the exerted compressive force allowed for the contact of SA and FEP. Thus, the electricity started to generate based on the electricity generation principle mentioned above. With the present SA-TENG, the single finger pressing motion can generate 200 V of *V*_OC_ and 60 µA of *I*_SC_. This amount of electricity is sufficient to directly light tens of the conventional light emitting diodes (LEDs) simultaneously (Figure 4b). The letters M, A, P, L, A and b, which are composed of parallel connected LEDs, can easily be lit. The harvested energy was stored in the conventional capacitor, which directly showed that the present SA-TENG could generate electricity from human body motion for the operation of the portable electronic devices. As shown in Figure 4c, the voltage of the capacitor (10 µF) reached 1.5 V within 8 s under vertical cyclic force with a magnitude of 8 N and a frequency of 10 Hz. Hence, the present strategy for the enhanced electrical output performance of TENG by using the M2M imprinting process is expected to widen the applicability of the TENG as a potent and practical energy harvester to power portable and wearable electronic devices.

## 4. Discussion

In this study, the steel stamps, which have nanometer or micrometer scale surface structures are successfully fabricated by using an fs laser. The fabricated steel stamps were then directly utilized in the M2M imprinting process to pattern the Al substrate, which can be directly utilized as one of the contact layers of the SA-TENG. The transcribed structures on the Al substrate were shown to play a role in enhancing the electrical output performance of the TENG through corresponding increased local pressure. By using the fabricated SA-TENG, the 200 V of *V*_OC_ and 60 µA of *I_SC_* were generated through single finger press motion. Given that the utilization of the M2M imprinting process enables rapid, versatile and easily accessible structuring of the metal substrate, which can be directly used as a contact layer on the TENG to significantly enhance its electrical output performance, the present study might significantly contribute to broadening the applicability of the TENG from a fabrication standpoint.

## Supplementary Materials

The following materials are available online at http://www.mdpi.com/2072-666X/9/11/551/s1, Table S1: M2M processing condition and design of experiment, Table S2: Nine representative cases of experiments in the M2M imprinting process, Table S3: *TQ*s in terms of conical microstructures, Table S4: *TQ*s in terms of line nanostructures. Figure S1: Close-up views of the *V*_OC_ and *I*_SC_ showing the detailed profiles of peaks. Figure S2: Result of numerical analysis to investigate the deformation of the contact surface during a contact situation. Figure S3: Experimental result showing strong mechanical durability of the present SA-TENG.
