# Supplementary material for: Development of the Triboelectric Nanogenerator Using a Metal-to-Metal Imprinting Process for Improved Electrical Output
Source: Micromachines (Basel). 2018 Oct 27;9(11):551. doi: 10.3390/mi9110551 (PMC6267296; doi:10.3390/mi9110551)
Supplement: Supplementary file 1 [file micromachines-09-00551-s001.pdf]

Supplementary Materials: Development of the Triboelectric Nanogenerator Using a Metal-to-Metal Imprinting Method for Improved Electrical Output

Moonwoo La, Jun Hyuk Choi, Jeong-Young Choi, Taek Yong Hwang, Jeongjin Kang and Dongwhi Choi

Effect of the processing condition of the metal to metal (M2M) imprinting on the transcription quality (TQ) of the structures on the steel stamp

The three parameters of the imprinting pressure (P_i), imprinting temperature (T_i) and holding time (t_i) are utilized as control parameters of the M2M imprinting process and they are varied at three levels (Table S1). We designed the experiment and as a result, nine representative cases were chosen and are shown in Table S2. The *TQ*s in terms of width (*W*), period (*P*) and Height (*H*) of the structures describing the features on the surface were measured.

The TQs in terms of conical microstructures are shown in Table S3 and it can be easily seen that case 6 had the highest averaged *TQ*, which represented the overall transcription quality of the structures on the stamp to the Al substrate. With the processing condition of case 9, the detachment of the substrate from the stamp failed due to the extreme processing parameters. From the analysis, the processing condition of P_i = 15 MPa , T_i = 200 °C, t_i = 1 min was utilized in this study to investigate the effect of the microstructures on the electrical output performance of the TENG.

The TQs in terms of line nanostructures are in Table S4 and it can be easily seen that case 9 has the highest TQ_{ave}. From the analysis, the processing condition of P_i = 25 MPa , T_i = 200 °C, t_i = 5 min was utilized in this study to investigate the effect of the nanostructures on the electrical output performance of the TENG.

Based on the aforementioned analyses, the optimized processing condition needs to be determined but falls outside the scope of this paper and thus it remains as future work.

Table S1. The M2M processing condition and design of the experiment.

Level	1	2	3
P_i (MPa)	5	15	25
T_i (°C)	25	120	200
t_i (min)	1	5	10

Table S2. Nine representative cases of experiments in the M2M imprinting process.

Case No.	Level of P_i	Level of T_i	Level of t_i
Case 1	1	1	1
Case 2	1	2	2
Case 3	1	3	3
Case 4	2	1	2
Case 5	2	2	3
Case 6	2	3	1
Case 7	3	1	3
Case 8	3	2	1
Case 9	3	3	2

Table S3. *TQ*s in terms of conical microstructures.

Case No.	TQ_W	TQ_P	TQ_H	TQ_{ave}
**Case 1**	59.8	70.6	34.0	54.8
**Case 2**	62.9	74.5	30.6	56.0
**Case 3**	78.2	80.2	96.6	85.0
**Case 4**	72.1	80.2	46.0	66.1
**Case 5**	79.1	88.1	75.7	81.0
**Case 6**	94.3	97.4	94.9	95.5
**Case 7**	85.0	87.6	80.4	84.3
**Case 8**	85.5	91.2	102.6	93.1
**Case 9**	N/A	N/A	N/A	N/A

Table S4. *TQ*s in terms of line nanostructures.

Case No.	TQ_W	TQ_P	TQ_H	TQ_{ave}
**Case 1**	42.7	84.7	24.0	50.5
**Case 2**	73.4	81.5	54.0	69.6
**Case 3**	53.7	99.1	61.6	71.5
**Case 4**	51.4	93.9	28.0	57.8
**Case 5**	55.1	98.4	38.2	63.9
**Case 6**	65.5	103.9	67.0	78.8
**Case 7**	74.1	103.9	58.9	79.0
**Case 8**	70.5	103.5	50.5	74.8
**Case 9**	79.9	99.8	85.2	88.3

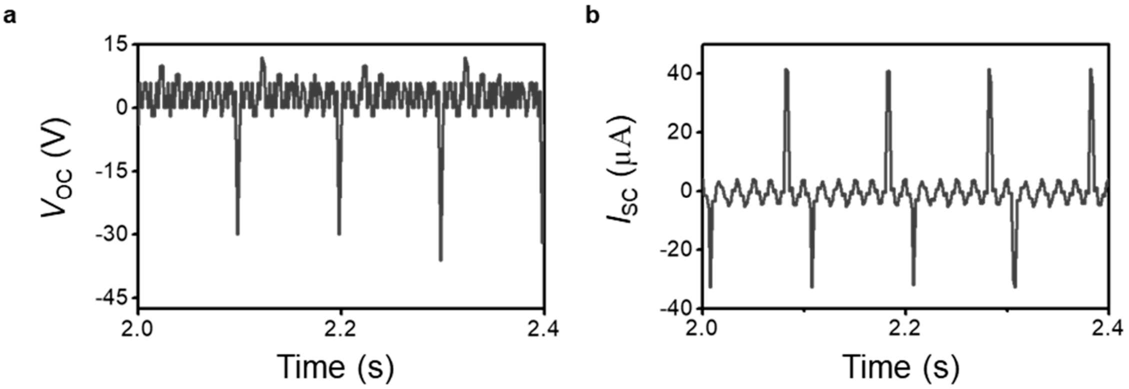

Figure S1. Close-up views of the V_{OC} and I_{SC} showing the detailed profiles of peaks.

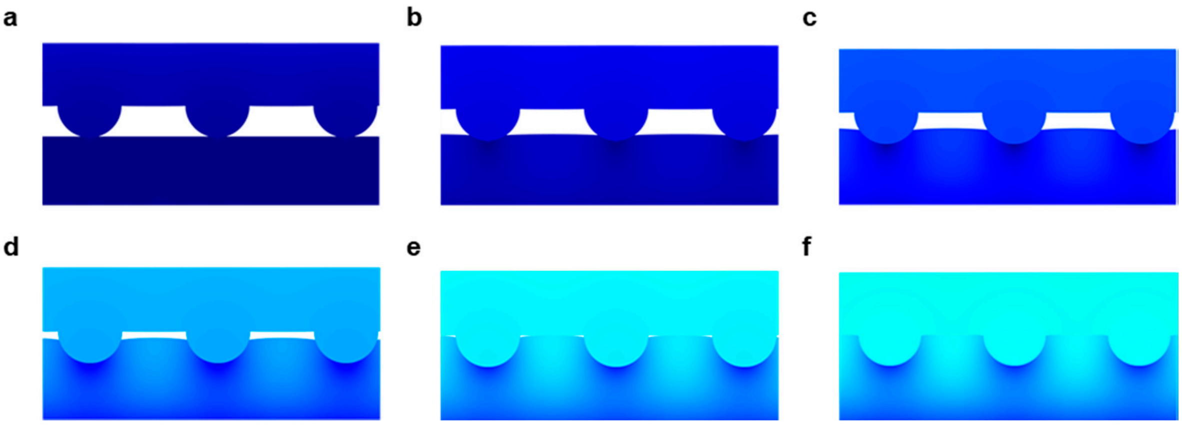

Figure S2. The result of numerical analysis to investigate the deformation of the contact surface during a contact situation.

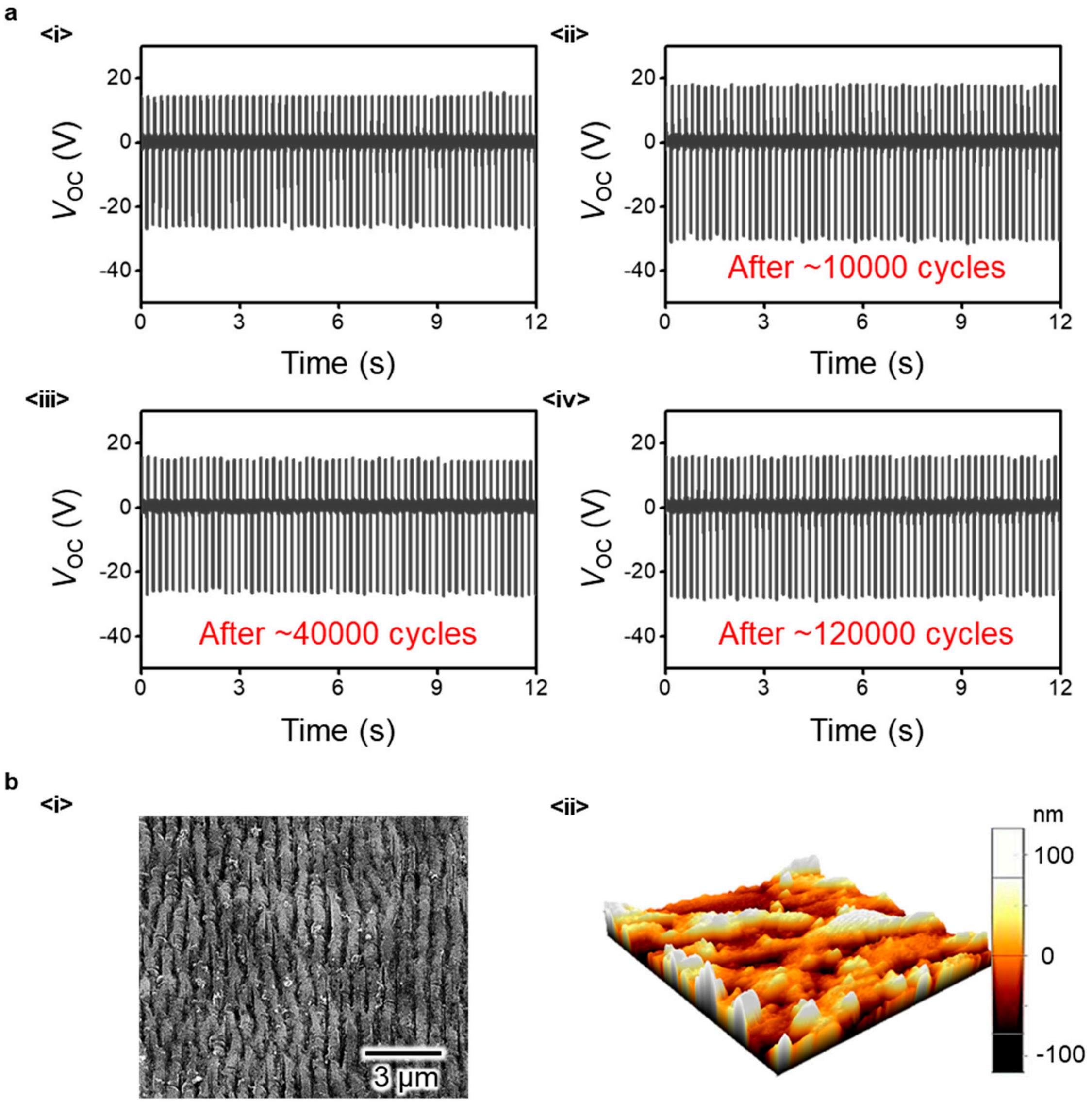

Figure S3. Experimental results showing strong mechanical durability of the present SA-TENG. (**a**) After an extremely large number of contact-separation working cycles (~10^6) by applying a force with a magnitude of 8 N, there was no degradation on the electrical output performance and (**b**) no significant changes in the surface structures with <i> SEM and <ii> AFM images.
